# Learning Handwriting: Factors Affecting Pen-Movement Fluency in Beginning Writers

**DOI:** 10.3389/fpsyg.2021.663829

**Published:** 2021-05-20

**Authors:** Camilla L. Fitjar, Vibeke Rønneberg, Guido Nottbusch, Mark Torrance

**Affiliations:** ^1^Norwegian Reading Centre, University of Stavanger, Stavanger, Norway; ^2^Human Science Faculty, Primary School Education, University of Potsdam, Potsdam, Germany; ^3^Psychology Department, School of Social Sciences, Nottingham Trent University, Nottingham, United Kingdom

**Keywords:** children, handwriting, fluency, pen-control, letter knowledge

## Abstract

Skilled handwriting of single letters is associated not only with a neat final product but also with fluent pen-movement, characterized by a smooth pen-tip velocity profile. Our study explored fluency when writing single letters in children who were just beginning to learn to handwrite, and the extent to which this was predicted by the children’s pen-control ability and by their letter knowledge. 176 Norwegian children formed letters by copying and from dictation (i.e., in response to hearing letter sounds). Performance on these tasks was assessed in terms of the counts of velocity inversions as the children produced sub-letter features that would be produced by competent handwriters as a single, smooth (ballistic) action. We found that there was considerable variation in these measures across writers, even when producing well-formed letters. Children also copied unfamiliar symbols, completed various pen-control tasks (drawing lines, circles, garlands, and figure eights), and tasks that assessed knowledge of letter sounds and shapes. After controlling for pen-control ability, pen-movement fluency was affected by letter knowledge (specifically children’s performance on a task that required selecting graphemes on the basis of their sound). This was the case when children retrieved letter forms from dictated letter sounds, but also when directly copying letters and, unexpectedly, when copying unfamiliar symbols. These findings suggest that familiarity with a letter affects movement fluency during letter production but may also point towards a more general ability to process new letter-like symbols in children with good letter knowledge.

## Introduction

It is still the case that in nearly all educational contexts children first learn to write by forming letters with pen or pencil on paper. The ability to handwrite is therefore a prerequisite for beginning to write. There is also evidence that, as children write longer texts, ability to retrieve and form letters and words quickly predicts the substantive quality of their written compositions (Feng et al., 2019). Several authors have argued that slow handwritten output not only reduces productivity – important when task duration is limited by time or motivation – but also demands attention that might otherwise be devoted to thinking about higher-level text structures (e.g., Berninger and Winn, 2006; Alves et al., 2016).

Although evidence suggests that handwriting ability is important for successful writing, relatively little is known about the factors that contribute to letter-formation fluency. Studies exploring correlation with text quality, such as those reviewed by Feng et al. (2019) measure handwriting ability using tasks that require reading and copying sentences (Barnett et al., 2009; Olinghouse and Graham, 2009) and/or written alphabet recall (Berninger and Rutberg, 1992; Kent et al., 2014). Successful performance of these tasks requires a broad combination of reading, orthographic, motor, and memory skills. Our present concern is more narrow. We focus specifically on the final, graphomotor components of the cascade of processes that comprise written production. van Galen (1991) describes this as occurring through a combination of allograph selection, size control, and muscle adjustment. These processes take as input an abstract letter representation (a grapheme) and end with the finger and arm movements that give the real-time trajectory of the pen across the page. The aim of the research that we report in this paper was to explore child level factors that predict fluent pen movement when forming letters.

The ability to produce fluent pen movement is, in principle at least, distinct from the neatness or accuracy of the resulting handwritten text. Consider the example in Figure 1. In all three cases, the final product is a well-formed (accurate) uppercase A. A classroom teacher looking to correct handwriting inaccuracy would pass over all three without comment. However, time taken to produce the highlighted feature by Writer C was four times longer than for Writer B, and over 10 times longer than for Writer A. The reason for this is clear from the velocity profiles. Whereas Writer A (an adult) produced the feature with a single acceleration and deceleration of the pen-tip, for Writers B and C, both children in early first-grade, pen movement involved multiple velocity inversions (acceleration and deceleration episodes).

**FIGURE 1:**
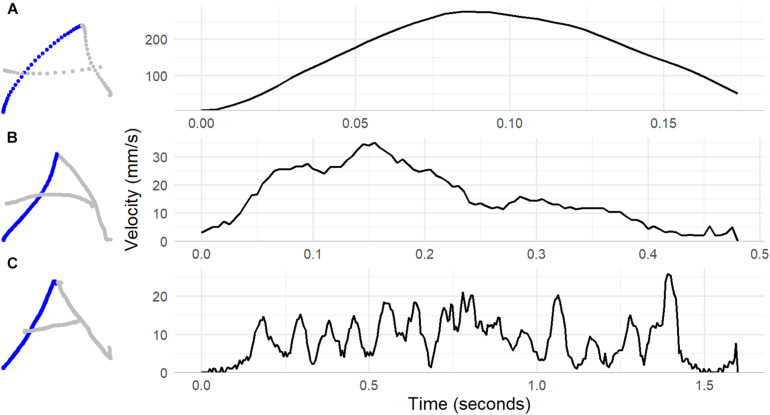
Pen trajectory and velocity profiles for the first feature of the letter A (shown in blue) written by an adult in panel **(A)**, and two first-grade children in panel **(B,C)**.

There is a developmental trend across primary school years from hesitant pen-movement in early years to smooth, automatic, and ballistic movements in later years (Chartrel and Vinter, 2008; Accardo et al., 2013). The main focus of previous research has been on comparison among children showing neat and untidy handwriting (van Galen et al., 1993; Rosenblum and Werner, 2006; Di Brina et al., 2008; Danna et al., 2013; Asselborn et al., 2018; Gargot et al., 2020). Di Brina et al. (2008) in a sample of second and third grade children examined the similarity in pen-movement trajectory across multiple repetitions of the same letter. Children who were categorised as dysgraphic based on that neatness of their handwriting showed substantially greater variability than students who were classified as good writers, suggesting that differences lie in the extent to which execution is based on stored motor plans. van Galen et al. (1993) used power spectral density analysis – an approach based in signal processing theory – compare children in grades 2–4 identified by their teacher as having poor handwriting to children who wrote neatly. They found that the pen movement of children with poor handwriting showed more power (more movement variation) at frequencies typically associated with movement tremor and less power at frequencies associated with intentional propulsive movement. Rosenblum et al. (2006) compared two groups on several temporal and velocity measures from the production of specific sub-letter features (the two strokes that form the single Hebrew letter ה). Children with teacher-identified dysgraphia were only slightly, and not significantly, slower to form features, but showed substantially more velocity inversions. Danna et al. (2013) compared similar groups on signal-to-noise velocity peaks difference (SNvpd). This is a measure that is conceptually similar to those derived from power spectral density analysis. SNvpd is the difference in counts of velocity peaks, across letters or words, detected after low and after higher waveband filtering. Peaks detected after low waveband filtering are assumed to be peaks that occur as part of fluent, ballistic movement, for example the single peak shown in the upper panel in Figure 1. Children with dysgraphia had SNvpd values over twice those of children within normal range handwriting accuracy. Similar measures based in velocity fluctuation discriminate handwriting in children with developmental coordination disorder (Chang and Yu, 2010).

There is, therefore, a relationship between handwriting accuracy (neatness) and the smoothness of the pen-tip speed profile, at least when comparing extreme groups. What is less clear is what underlying abilities predict handwriting ability. Del Giudice et al. (2000) found that accuracy in copying non-letter patterns and figures develops rapidly between the ages of 4 years 6 months and 5 years (20% accuracy to 80% accuracy on a shape copying task) in a sample of children attending kindergarten. There is evidence that shape copying in turn predicts letter copying accuracy in kindergarten (Weil et al., 1994; Marr et al., 2001). Shape copying in kindergarten may (van Hartingsveldt et al., 2015) or may not (Marr and Cermak, 2002) predict letter-writing accuracy in first grade. There is also some evidence that letter knowledge–knowledge of letter shapes and sounds–predicts letter-formation accuracy. Molfese et al. (2011) found that handwriting accuracy in kindergarten correlated with letter and word naming. For 8-10-year-olds, Caravolas et al. (2020) found that spelling ability predicted neatness of letter formation.

Accuracy when forming letters is therefore correlated both with domain-general graphomotor skill and with letter knowledge, as might be expected. Our present purpose is to examine the extent to which these factors affect pen-movement fluency. It would seem very probable that graphomotor skill, based on measures that require pen-control when producing non-letter figures, generalises to the production of letters. The main question addressed in the research that we report in this paper is whether, after control for graphomotor skill, children’s general letter knowledge predicts within-letter pen-movement fluency. Specifically we asked whether in situations where children are, for example, copying a letter – i.e., they have a representation of the shape that they aim to produce – general letter-level knowledge, as measured by, for example, the ability to map between phonemes and graphemes, predicts movement fluency. The answer to this question is less straightforward. It may be that retrieval of the letter form – the output from the allograph-selection module in van Galen’s architecture for models of handwriting (van Galen, 1991) – is always complete before production of a letter is initiated. If this is the case then we would not expect within-letter pen movement fluency to be affected by letter-knowledge. Alternatively, it may be that, in early writers in particular, letter-knowledge continues to affect movement once pen movement has started, either because they continue to plan the shape that they are forming while the pen in moving, or because allograph knowledge informs the control processes (Meyer et al., 1988; Glover, 2004) that are engaged after movement has been initiated.

### Present Study

The study we report in this paper explored predictors of letter-level handwriting fluency in children who were just beginning to learn to handwrite. Our concern was specifically with the final graphomotor components of the cascade of processes that comprise written production. Our focus, therefore, was specifically on a child’s ability to move the pen fluently and accurately on the page to create the form of a known letter.

We addressed two questions. In children at the beginning stages of learning to write…

(1) To what extent do factors associated with pen-control and with letter knowledge affect pen-tip movement fluency in copied letters and symbols?
(2) After control for letter-copying ability, to what extent do factors associated with letter knowledge affect fluency when forming letters from dictation (i.e., in response to hearing letter sounds)?

We hypothesised, uncontroversially, that handwriting performance would in part depend on pen-control ability (i.e., we hypothesised that ability to fluently reproduce specific pen movements would transfer to the fluent production of letter forms). We also tested the prediction that, after control for graphomotor (pen-control) ability, the extent to which letter features were produced fluently would be dependent on a child’s general abstract letter knowledge. This prediction was tested in a character-copying task by the inclusion of unfamiliar symbols as controls. If letter knowledge affects fluency then this effect will be present when children are drawing letters but not when they are producing unfamiliar symbols. Including letter- and symbol-copying performance as covariates in analysis of writing-to-dictation fluency allowed us to isolate effects specifically associated with retrieving letter-form from memory.

## Materials and Methods

### Design and Participants

We report data from 176 first grade children from 10 Norwegian schools who completed various tasks: copying characters, writing letters to dictation, controlling the pen and various letter knowledge measures. Of the 187 children whose parents gave permission, handwriting data from nine children were corrupted and two children were unable to complete any tasks. The children included in the study were on average 74.6 months (6.2 years). There were 90 boys and 86 girls.

### Educational Context

In Norway, first grade is the first encounter with formal literacy instruction. Children start first grade in August the year of their sixth birthday. Before they start school, 97.6% of all Norwegian 5-year-olds attend Kindergarten (The Norwegian Directorate for Education and Training, 2020). There is no curriculum with learning goals for the Norwegian kindergarten, but there is a framework that stipulates that children should be encouraged and supported in using language to communicate (The Norwegian Directorate for Education and Training, 2018). Consequently, children enter school in Norway with no formal instruction in either letter knowledge or pen-control.

### Equipment and Procedure

Children were tested over 2 days within 4 weeks of school entry. Day 1 was dedicated to testing letter knowledge. Day 2 was dedicated to collecting handwriting and pen-control data. Each child was invited to join the researcher to do the tasks in a quiet room at the school. Each session lasted approximately 20 min. All handwriting and pen-control data were collected with Wacom Intuos XL digitising tablets and HP Elitebook i5 laptops. Pen-tip location was sampled at intervals of around 7.5 ms (133 Hz) and with a spatial resolution of at least 330 lines/cm. An A3 sized paper test-sheet was secured to the tablet and the children wrote with an inking ballpoint stylus. The children were first asked to draw with the pen on the paper and to write their name to familiarise themselves with the equipment. They then completed the pen-control tasks, the copy task, and finally the letter-to-dictation task. Software for pen-movement capture and analysis was provided by the OpenHandWrite suite of programs (Simpson et al., 2021) which provide a digitising tablet interface for PsychoPy (Peirce et al., 2019).

### Measures

#### Copying Task

Children were asked to copy once, in pre-printed boxes of 2.5 × 2.5 cm, each of the following characters: Ø Ω A ǂ M d Ψ h T Ɣ e ゐ g R. The researcher showed the child one item at the time, printed on paper (7 cm by 5 cm). The child was then told to write the letter, even if they did not recognise it as we told them there were some “silly” letters as well (these were the non-letter symbols). The child was shown one letter, <Ø>, and one symbol, <Ω>, as practice items. An example pen-trace from this task can be found in Figure 2.

**FIGURE 2:**
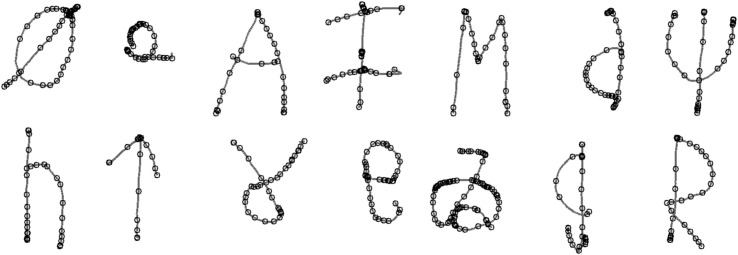
Example output from the character copying task, produced by a child with relatively disfluent handwriting. Small circles represent the location in the pen trace of a velocity peak, after 10 Hz filtering.

After data collection the first and second author manually marked-up the digitised trace of each character produced by children in the copying and dictation tasks. This involved segmenting characters into composite features, identifying temporal and spatial start and end points for each feature. This permitted subsequent analysis of pen-tip velocity profile for the child’s production of that feature. Each feature was also coded for accuracy.

##### Feature coding

Segmentation of characters into features was determined by a strict (objective) coding scheme. We identified features as components of characters that competent handwriters would typically produce with a single pen-stroke. Feature definitions were entirely spatial (i.e., defined the shape of the completed feature) and coded independently of information concerning the pen movement with which they were produced. Each character was decomposed into a unique set of curved and / or straight-line features. For example, character > *A* < was decomposed into three straight-line features, the character <Ø> was decomposed into a straight line and a closed curve, and so forth. We allowed for different allographs. The character > *A* < could legally also be composed of a single open curve as a replacement for the two diagonal uprights.

Segmented and coded in this way the target characters represented a total of 20 features in letters (14 straight, six curved) and 12 features in non-letter symbols (eight straight, four curved).

Once a feature was identified as present (i.e., could be matched to a feature of the target character), it was coded as either accurate or inaccurate. Our coding scheme was rule based and broadly followed (Reisman, 1993), but defined acceptable feature forms in terms of size, curvature, slope, and curvature relative to other features. A feature was coded as malformed (inaccurate) if it deviated beyond parameters defined as an acceptable representation of an allograph of the target letter. For example the leftmost upright of an uppercase > *A* <, when formed with two diagonal uprights (the feature identified in Figure 1) was coded malformed if it deviated from the following: Meets the second upright at an acute angle of between 20 and 90 degrees not deviate from straight by more than 1/6th of its length, does not deviate in length by more than 1/6th of the length of the second upright, and meets the second upright with separation or overlap of not more than 1/6th of its length. Our coding manual is publicly available (see data availability statement for access).

##### Feature production fluency

We calculated tangential velocity of the pen-tip at each sample point, and filtered the resulting velocity timecourse with a 10 Hz fourth order low-pass Butterworth filter to remove measurement noise. We then counted remaining velocity maxima for each feature. Features were defined such that competent production could be assumed to be associated with a single velocity maximum (features could be produced with a single pen-stroke). If a child’s pen movement when producing the feature was less than fluently, then this would be associated with one or more additional velocity maxima (illustrated in Figure 1 above). The production of each feature was, therefore, given a disfluency score corresponding to a count of the number of velocity maxima associated with its production.

In pilot data collected using the same measures with adults, currently being prepared for publication, modal number of velocity peaks were one for straight features and two for curves. This provides support for our claim that features in our coding scheme represented letter components that competent handwriters would typically produce in a single ballistic action.

Distribution of this fluency measure, and relationship, at a feature level, with mean velocity, trace length, and duration, are reported in the Appendix in Figures A1, A2 respectively.

#### Dictation Task

In the dictation task the children heard letter-sounds, one at the time, and were asked to write the corresponding letters in pre-printed boxes of 2.5 × 2.5 cm. No instructions were given on how to write the letters (e.g., upper or lower case) and children were told to write the letter as they normally would. The first two sounds were pronounced by the researcher to ensure the child understood the task, then followed nine sounds played by the computer. The first computer-sound was excluded from the analyses as this was more likely to be affected by technical difficulties. The letter-sounds included in the analyses are /l/, /f/, /i/, /b/, /o/, /p/, /u/, /s/, /k/, and /v/. These letters were selected as producing them demands similar motor plans regardless of whether the child chose to write upper or lower-case versions, and therefore the production processes can be compared. That would not be the case for > *e* < and > *E* <, which clearly demand different motor plans.

To be identified as an attempt at the target letter in the dictation task, all features associated with the target had to be present, though they could be badly shaped, sized or positioned. Letters identified as successful attempts were then coded for accuracy and fluency measures using the same procedures as for the copying task.

#### Pen-Control Tasks

**FIGURE 3:**
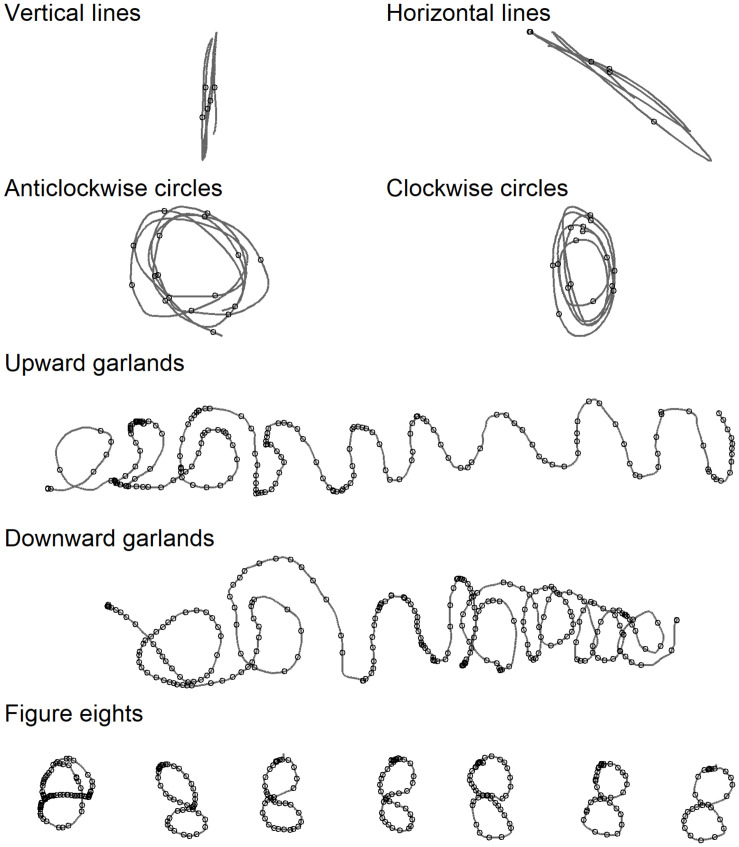
Example output from the various pen-control tasks, produced by the same child as in Figure 2. Small circles represent the location in the pen trace of a velocity peak, after 10 Hz filtering.

The pen-skill tasks were seven tasks aimed at measuring the child’s ability to control the pen. The tasks were adapted from tasks used by Gerth et al. (2016). Example pen traces from these tasks are shown in Figure 3.

##### Straight lines

The tasks were first to draw overlapping horizontal and next overlapping vertical straight lines repeatedly without lifting the pen. The researcher first modelled the pen action, which the child was then asked to reproduce. The children were not stopped until they had produced 10–15 up and down movements or back and forth movements, respectively. The first two lines produced were dropped from analysis. Fluency measures were then taken from the next five consecutive lines made without any pen lifts.

##### Circles

The tasks were to draw overlapping clockwise and anti-clockwise circles, in each case with a continuous pen movement and with between 10 and 15 repetitions, keeping within a printed box (5 × 7 cm). The researcher first modelled the pen action. The initial two repetitions going in the correct direction were dropped from analysis. Fluency measures were then taken from the next five consecutive circles that were made without any pen lifts. A circle was considered successful if it mainly consisted of one curved line surrounding an open space, with no line crossings before the circle was complete. Round, more egg-shaped, shapes, were accepted.

##### Garlands

The tasks were to produce first an upward pointing and then a downward pointing garland in a continuous pen movement. The researcher first showed a printed sample of garlands and then modelled the pen action. Each garland was drawn on a pre-printed line (17 cm) with a 4.8 cm gap up to the previous task. The children were encouraged to keep going until they had produced at least ten loops. Fluency measures were extracted across all pen movement during the tasks^1^.

##### Figure eights

The researcher first showed a printed sample of the figure eight and then modelled the pen action required to draw a figure eight as one continuous movement. Children then attempted to reproduce this seven times, drawing each within a separate box (2.5 × 2.5 cm). The fluency measure represents the sum of all the figure eights a child produced.

The letter features analysed in the copy and dictation tasks have required a small, fixed number of necessary velocity peaks for their production [see, for example, Chartrel and Vinter (2008)]. In the garlands task, however, children varied in how much they produced and in the shape of their output. To control for, this fluency was measured with Signal-to-Noise velocity peaks difference (SNvpd) following the method described by Danna et al. (2013). Following Danna et al., we counted velocity peaks after 5 Hz and after 10 Hz filtering and report the difference. For consistency we also used SNvpd as a measure of fluency on the other three tasks.

#### Letter Knowledge

Tasks were taken from a battery of tests standardised for use with Norwegian first grade children (Lundetræ et al., 2017; Solheim et al., 2017, 2018).

##### Phoneme to grapheme encoding

Children heard letter-sounds played on a tablet computer, and then saw four upper-case letters. The children were asked to find and press the letter that corresponded to the sound. Children completed 24 trials, one for each letter of the Norwegian alphabet, excluding *C*, *Q*, *X*, *Y*, and *Z* which are rarely used, with distractor letters chosen randomly. Each trial was scored correct (1) or incorrect (0), and the maximum score was 24.

##### Grapheme to phoneme decoding

Children saw a lower-case letter on the tablet screen and were asked to speak the letter sound. Children giving letter names were prompted for letter sounds. The target letters were the same as in the previous task. Each trial was scored correct (1) or incorrect (0), and the maximum score was 24.

##### Phoneme isolation

This was a partial phonological segmentation task in which the children were shown a picture of an object, the researcher named the object, and the child was then asked for the first sound of the name (e.g., “Dette er en bok. Hva er den første lyden i bok?≫/“*This is a book. What is the first sound in book?”*). The task terminated after two consecutive failed attempts. Each trial was scored correct (1) or incorrect (0), and the maximum score was 10.

##### Phoneme blending

The children were shown pictures of four objects or actions and a pre-recorded voice named each word: ri/*ride*, ris/*rice*, ring/*ring*, and rips/*redcurrant* (a high-frequency word for Norwegian children). The pre-recorded voice told the child to press the image corresponding to /r/ /i/ /s/. The children had to blend the sounds to make the word. The task terminated after two consecutive failed attempts. Each trial was scored correct (1) or incorrect (0), and the maximum score was 8.

## Results

We evaluated the effects of pen-control and literacy-skill measures on character-writing fluency by comparing a sequence of nested linear mixed effects models (e.g., Baayen et al., 2008), implemented in the lme4 R package (Bates et al., 2015). Our data comprised observations of fluency and accuracy for each character feature drawn by each child. Observations were therefore nested within item (the character) and within child. All models therefore included random by-item and by-child intercepts. Model comparison was by likelihood ratio χ^2^ test. Statistical significance for parameter estimates for models with continuous outcomes was established by evaluating against a *t* distribution with Satterthwaite approximation for denominator degrees of freedom (implemented in lmerTest; Kuznetsova et al., 2017). For generalised linear models, with dichotomous outcomes, we evaluated against a *z* distribution.

Descriptive statistics for predictor variables (means and bivariate correlations) can be found in Table 1. As might be expected, we found strong correlation between our grapheme-to-phoneme decoding and phoneme-to-grapheme encoding ability measures. To avoid issues with collinearity, only the encoding measure was retained on the grounds that this ability is more likely to be causally implicated in grapheme production fluency.

**TABLE 1:** Mean scores and bivariate correlations among letter-knowledge, pen-control, and copying letters and symbols fluency measures.

	**Mean (SD)**	**Isolation**	**Blending**	**Encoding**	**Decoding**	**Lines**	**Circles**	**Garlands**	**Eights**	**Copy letters**
Phoneme isolation	6.2 (3.6)									
Phoneme blending	3.5 (2.5)	0.56								
Phoneme to grapheme encoding	18 (5.6)	0.59	0.51							
Grapheme to phoneme decoding	11 (6.8)	0.69	0.60	0.72						
Pen-control: Lines	0.46 (1.4)	−0.07	−0.08	0.01	−0.04					
Pen-control: Circles	6.7 (5.8)	−0.11	−0.26	−0.11	−0.09	0.40				
Pen-control: Garlands	61 (41)	0.09	0.02	0.04	−0.00	0.18	0.24			
Pen-control: Eights	98 (52)	0.00	−0.00	−0.12	−0.16	0.04	0.04	0.30		
Copy-fluency: Letters	10 (4.3)	−0.16	−0.13	−0.32	−0.30	0.09	0.20	0.19	0.41	
Copy-fluency: Symbols	13 (5.8)	−0.03	−0.02	−0.16	−0.19	0.05	0.16	0.26	0.45	0.70

**p* < 0.001 for | *r*| > 0.25. For pen-control, measures are SNvpd values across the entire task. For copy fluency, values are for mean number of velocity peaks (10 Hz filtering) per feature, averaged within and then across participants.*

We first give findings from analyses examining factors affecting character copying, and then findings from analyses examining factors affecting writing letters to dictation. In each case our main focus is on factors that affect the extent to which children’s pen-tip movement is fluent.

### Letter and Symbol Copying

In this section, we explore item-level and child-level factors affecting character copying fluency, measured as the number of velocity peaks in the pen-movement associated with each feature. We started with an intercept-only model, and then added fixed effects incrementally, starting with factors associated with features – Model 1 adds whether or not the feature was correctly formed, and Model 2 adds whether it was a curve. Model 3 adds a fixed effect for whether the character being produced was a letter or symbol. Model 3a adds child age, and Model 4 adds the four pen-control measures. We then explored whether the effects of pen-control measures were moderated by whether the feature being produced was a curve or straight line (Model 5) and whether or not the character was a letter (Model 6). Finally, we added fixed effects for the three letter knowledge measures (phoneme to grapheme encoding, phoneme isolation, and phoneme blending (Model 7) and explored whether these effects were moderated by whether the target being produced was a letter or a symbol (Model 8).

Table 2 details models and model-comparison statistics. Each subsequent model provided better fit, with three exceptions: We found no effect of whether the target was a letter, although this factor was included in subsequent models to permit accurate interpretation of subsequent interaction effects. We also found no effect of child age, or of interaction between letter knowledge measures and whether or not the character was a letter. These factors were omitted from the final model. The best-fit model was, therefore, Model 7. This gave an estimated marginal *R*^2^ of 0.18 (Nakagawa and Schielzeth, 2013), and intra-class correlations of 0.23 for random effects of child and 0.15 for random effects of item.

**TABLE 2:** Model comparison for models predicting pen-movement disfluency (velocity peak count) in the character copying task.

	**Fixed factor(s) added**	***X*^2^, d*f*, *p***
Model 1	Feature malformed (vs. correct)	100, 1, <0.001
Model 2	Target feature is a curve (vs. straight line)	420, 1, <0.001
Model 3	Character is a letter (vs. symbol)	2.2, 1, 0.136
Model 3a	(Child age)	0.09, 1, 0.77
Model 4	Pen-control measures	52, 4, <0.001
Model 5	Interactions between pen-control measures and whether the target feature is a curve	35, 4, <0.001
Model 6	Interactions between pen-control measures and whether the character is a letter	13, 4, 0.013
Model 7	Letter-knowledge measures	10, 3, 0.017
Model 8	Interactions between literacy-ability measures and whether the character is a letter	4.2, 4, 0.24

*Models were nested, with all fixed factors added at a particular stage carried forward to subsequent model, with the exception of age (Model 3a). Model 1 was compared with an intercept-only model. Model 7 is the best fit model.*

**FIGURE 4:**
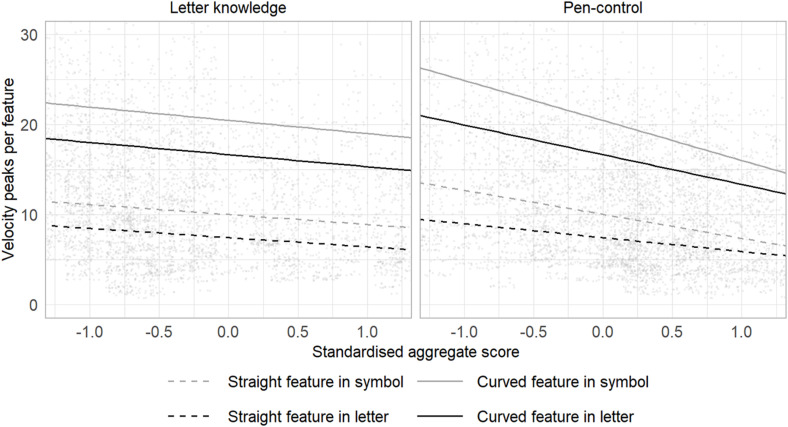
Letter knowledge and pen-control measures as predictors of letters and symbols copying fluency (velocity peaks). Parameter estimates from best-fit model. X-axis values are for just those measures showing statistically significant effect: Letter knowledge is standardised phoneme-to-grapheme encoding score. Pen-control is the reversed mean of standardised fluency measures for garlands and figure-eights. Points represent raw observations.

Parameter estimates from the best-fit model are given in Table 3. These indicate the following: (a) when a child produced a badly formed feature, this tended to also be associated with low fluency, (b) curved features were produced less fluently than features comprising straight lines, (c) children’s fluency when producing continuous garlands, and particularly figure-eights, predicted character copying fluency, (d) the effect of figure-eight performance on copying fluency was particularly strong when the feature being produced was a curve, (e) effects of pen-control measured by the garlands and figure-eight tasks was slightly greater when copying symbols than when copying letters, and (f) children who performed well on the phoneme to grapheme encoding task showed greater letter and symbol-copying fluency. These findings are illustrated in Figure 4.

**TABLE 3:** Pen-movement disfluency (velocity peak count) when copying characters.

	**Main effects**	**Interaction with Feature-is-curve**	**Interaction with Character-is-letter**
Intercept	10 [8.0, 12]		
Feature is malformed (vs. correct)	3.0 [2.1, 3.9]***		
Feature is a curve (vs. straight line)	7.3 [6.7, 8.0]***		
Character is a letter (vs. symbol)	−2.1 [−4.8, 0.52]		
Pen−control fluency			
Lines	−0.31 [−1.0, 0.42]	0.12 [−0.46, 0.70]	0.32 [−0.24, 0.87]
Circles	0.55 [−0.20, 1.3]	0.53 [−0.06, 1.1]	−0.06 [−0.63, 0.50]
Garlands	0.73 [0.01, 1.5]*	0.37 [−0.21, 0.94]	−0.56 [−1.1, −0.00]*
Figure eights	1.8 [1.1, 2.5]***	1.3 [0.70, 1.8]***	−0.58 [−1.1, −0.05]*
Letter knowledge			
Phoneme to Grapheme encoding	−1.1 [−1.8, −0.34]**		
Phoneme isolation	0.01 [−0.74, 0.75]		
Phoneme blending	0.36 [−0.35, 1.1]		

Estimated effects with 95% CI. Parameter estimates from a linear mixed-effects model with random by-item and by-subject intercepts. Blank cells indicate that effect was absent in the best-fit model. *p < 0.05; **p < 0.01; ***p < 0.001.

Interpreting findings related to the fluency of features that were produced accurately compared to those that were malformed requires understanding of how this relates to the shape of the feature and to whether the figure being produced was a letter or a non-letter symbol. Observed proportion of features malformed were as follows: Letters: straight features, *M* = 0.04, *Mdn* = 0.00, IQR [0.00, 0.07]; curved features, *M* = 0.13, *Mdn* = 0.14, IQR [0.00, 0.17]. Symbols: straight features, *M* = 0.09, *Mdn* = 0.00, IQR [0.00, 0.12]; curved features, *M* = 0.32, *Mdn* = 0.25, IQR [0.00, 0.50]. To explore the relationship among these factors we evaluated logistic generalised linear mixed effects models predicting whether or not a feature was formed correctly, starting with an intercept-only model (Model 0), and then adding dummy variables representing whether the feature was straight or curved (Model 1), whether the feature was part of a letter or a symbol (Model 2), and then their interaction (Model 3). We found evidence for an effect of feature shape, with curves produced less accurately [Model 1 vs. Model 0, χ^2^(1) > 100, *p <* 0.001], and some evidence that symbols were produced less accurately than letters [Model 2 vs. Model 1, χ^2^(1) = 4.46, *p* = 0.035]. There was no evidence of an interaction between these factors.

### Writing Letters to Dictation

Two children failed to retrieve any letters in the letter-writing to dictation task. These children are therefore omitted from this analysis. For the remainder of children, the median number of correct responses (responses that were identifiable as the target letter but may have included one or more malformed features) was 5 (IQR [4,8]) out of a maximum of 10. The analyses that follow are just of data from correct responses. In the resulting sample, the mean number of straight features included for each child was 7.82 (*SD* = 3.15) and curved features, *M* = 5.38, *SD* = 1.58.

We explored whether children’s letter knowledge predicts production fluency, over and above variance explained by children’s performance on the letter-copying task as follows: We started with an intercept-only model, and then added a dummy variable to control for whether or not the feature was malformed (Model 1). We then added measures of letter-copy and symbol-copy fluency, taken from the letter-copying task and aggregated within child (Model 2). Finally, we added the three letter knowledge measures (Model 3). We performed this analysis separately for straight and curved features. For straight features, each subsequent model provided better fit [χ^2^(1) = 10, *p* = 0.001; χ^2^(2) = 26, *p* < 0.001; χ^2^(3) = 10, *p* = 0.017, respectively]. Model 3, the best fit model, gave an estimated marginal *R*^2^ of 0.10, and intra-class correlations of 0.38 for random effects of child and 0.06 for random effects of item. For curved features, Models 1 and 2 both improved fit [χ^2^(1) = 4.5, *p* = 0.034 and χ^2^(2) = 80, *p* < 0.001] but we found no evidence of an effect of letter knowledge [Model 3, χ^2^(3) < 1]. Estimated marginal *R*^2^ was 0.13 for Model 2, the best fit model, with intra-class correlations of 0.13 for random effects of child and 0.21 for random effects of item.

Parameter estimates from the best-fit models are given in Table 4. Effects of letter and symbol-copying ability were similar for both straight and curved features. As might be expected, lack of fluency in copying was associated with lack of fluency when producing letters that were retrieved in response to their sounds, although this effect failed to reach significance for symbol copying as a predictor of straight feature production. The production of curved features was generally less fluent, as was the case for the letter and symbol-copying tasks but we found no evidence for effects of children’s letter knowledge. There was some evidence of effects of letter knowledge for straight features over and above variance explained by children’s performance on the letter-copying task. As was the case with copying, good performance on the phoneme to grapheme encoding task was associated with more fluent production. However, phoneme blending showed the reverse effect. We suspect that this is a statistical artefact resulting from relatively strong correlations among our letter knowledge measures, rather than representing a true effect.

**TABLE 4:** Parameter estimates from models predicting disfluency (velocity peak count) when children wrote letters to dictation.

	**Straight features**	**Curved features**
Intercept	6.6 [5.4, 7.7]	10 [7.9, 12]
Feature is malformed (vs. correct)	5.2 [1.8, 8.7]**	2.0 [0.12, 3.8]*
Symbol copying fluency	0.97 [−0.24, 2.2]	1.0 [0.37, 1.7]**
Letter copying fluency	1.5 [0.23, 2.7]*	1.8 [1.1, 2.5]***
Phoneme to Grapheme encoding	−1.2 [−2.3, −0.02]*	
Phoneme isolation	−0.95 [−2.1, 0.22]	
Phoneme blending	1.2 [0.06, 2.3] *	

Parameter estimates from a linear mixed-effects model with random by-item and by-subject intercepts. Blank cells indicate that effect was absent in the best-fit model. *p < 0.05; **p < 0.01; ***p < 0.001. All predictor variables were standardised, with the exception of the dummy variable representing malformed features.

When writing letters to dictation, the children who managed to reproduce the target letter tended to include all features in the correct shape, position and size, with 157 children (90%) making no errors on straight features, and 137 (79%) making no errors on curves.

## Discussion

Our analysis focussed on fluency of production of letter features, in beginning writers, that skilled adult handwriters would typically produce in one smooth, ballistic movement. We found that children in our sample typically produced these features disfluently, with multiple velocity inversions where skilled performance would result in only one or two. This is as might be expected given the lack of pre-school training in handwriting in the Norwegian educational system. Chartrel and Vinter (2008), using a velocity peak measure very similar to the one used in this study, found rather greater letter-copying fluency in children in the last year of French kindergarten. Curved features were produced less fluently than straight features, in both the copying and dictation tasks. In the relatively rare cases where a feature was malformed, these tended to also be produced with less fluency, again in both tasks.

We found, again as might be expected, that pen-control ability, measured by fluency when producing garlands and figure-eights, predicted fluency when copying characters. This effect was somewhat greater for curved features, and when copying non-letter symbols. Children with good letter knowledge, and specifically phoneme-to-grapheme encoding ability, copied both letters and symbols with greater fluency. When writing-to-dictation, with statistical control of letter-copying fluency, phoneme-to-grapheme encoding predicted fluency for straight features but not for curved features.

We first discuss effects of pen control and then effects of letter-knowledge. Fluency in the garlands and figure-eights tasks independently predicted character copying fluency, but fluency in the straight line and circles pen-control tasks did not. This was, we believe, for one or both of two reasons. First, these tasks did not discriminate between children in our sample. Mean number of super-numerous velocity peaks–velocity inversions that would not be expected in a handwriting–was roughly one per circle, when children drew circles, and were largely absent when children drew straight lines. This probably simply reflected the developmental stage of our sample. Although they had had little or no formal training in handwriting prior to data collection, at a mean age of 6.2 years their motor development and hand-eye coordination is likely to have been relatively advanced. Garlands and figure eights were substantially more challenging tasks for reasons including the fact that both figures include inflection points at which the direction of curvature changed. Second, drawing garlands and, particularly, isolated figure eights is not only a more complex skill but one that is closer to the specific abilities required to project letters and letter-like symbols. In both cases the pen movement was first presented to the child. However, we suspect that, unlike the repeated movement required for the lines and circles tasks, both of these tasks made direct demands on graphomotor skills (the ability to take a mental representation of a figure and reproduce it on the page).

Letter-knowledge, specifically performance on a phoneme-grapheme encoding task, also predicted pen-movement fluency. This is, perhaps, a more surprising finding. Existing models of handwriting production assume that grapheme and, in fact, allograph selection is complete before the movement to form a letter starts, even in early writers (van Galen, 1991; Pagliarini et al., 2017). Letter knowledge might, therefore, affect latency prior to starting a letter, but not movement fluency while the letter features are being drawn. There is, however, evidence that for children with a specific cognitive literacy deficit (dyslexia but not dysgraphia) the rhythmic nature of handwritten word production, that is present in even young children, breaks down (Pagliarini et al., 2015). This could be interpreted as suggesting that, at least in extreme cases, difficulty with mapping between graphemes and phonemes can result in pen-movement disfluency within letters rather than hesitation between letters or words.

Therefore one possible explanation of the association between letter-knowledge and within-feature fluency was that lack of knowledge directly interferes with production, either because motor planning is not complete at start-of-movement or because uncertainty activates control processes that then modify the planned action (Glover, 2004). This account does not, however, explain the fact that effects were present not only when participants were forming letters, but also when copying non-letter symbols. We suggest two further explanations. It may be that a precursor to developing good knowledge of phoneme-grapheme correspondence is the visuo-spatial ability to process novel letter-like shapes. This ability, in turn, is likely to increase fluency when copying unfamiliar symbols. This will particularly have been a factor if some children interpreted the copying task as requiring exact reproduction of the allograph with which they were presented, which will have necessarily been the case for characters that they did not recognise. A third possibility is that the direction of causality is reversed. Students who are able to handwrite fluently will be more productive. Practicing forming letter by hand may result in improved abstract letter knowledge (Longcamp et al., 2008; Bara and Bonneton-Botté, 2018; but see Bara et al., 2016), although this is more likely to occur when children have been exposed to formal, classroom writing instruction, which was not the case for our sample. The three explanations that we have offered are not mutually exclusive, and all three mechanisms may have been at play in our study. Future research could usefully aim at isolating these different effects.

Effects on fluency when forming letters in response to dictation were dependent on whether or not the letter feature was curved. For curved features fluency was predicted just by fluency on the letter copying task, with effects both for symbol copying and for letter copying. This suggests that, for curves–which were generally less fluently produced and therefore more graphomotorically demanding, performance was overdetermined by pen control ability. For straight lines residual variance was explained in part by letter-knowledge. As for the copy task we found that fluency was greater for children with better phoneme-to-grapheme encoding ability. However, we found that children who performed well on the blending task–an ability that requires phonological skill but not grapheme recognition–were less fluent, after control for the other two letter-knowledge variables. We do not have a straightforward explanation for this effect.

Finally, it is worth noting that, unlike studies with older children (e.g., van Galen et al., 1993) we did not find evidence of a trade-off between fluency and accuracy. In the relatively rare cases where the children in out sample produced letter features that were badly formed these tended to also be produced less fluently. Once some level of automaticity has been achieved then children have the flexibility to jettison some of control in the interests of writing with greater fluency, and therefore greater speed. However, our data suggests that the majority of children in our sample were not at a stage where they had the option to produce letter features with these less controlled, ballistic actions.

In summary, therefore, our paper presents a first attempt at unpacking factors that predict within-letter pen-movement fluency in beginning writers. As such, we have started to explore one of a number of components that contribute to transcription fluency as measured, for example, by speed of sentence copying (e.g., Barnett et al., 2009). A number of previous studies have demonstrated that children with untidy handwriting produce pen strokes with multiple velocity inversions, indicating a lack of automaticity and the need for ongoing motor-planning and correction after movement has been initiated. Our study started from the observation across children who correctly and neatly form letters–children who would not be identified by teachers as having difficulty with handwriting–there is considerable variation in fluency. Neat handwriting may therefore mask disfluency that has knock on effects for productivity and, perhaps, composition quality. Our findings indicate that disfluency is associated not just with weaker graphomotor (pen-control) ability but also with more general abstract letter knowledge. It would be premature to draw implications for instruction based on these findings. However, we recommend that future research gives attention to stroke fluency, alongside more macro-level fluency measures, in seeking to understand how children develop the complex cascade of processes that combine to permit fluent written composition.

## Data Availability Statement

Data, scripts for statistical analysis and manual for coding letter features from this study are available at: https://doi.org/10.17605/OSF.IO/P8JBF.

## Ethics Statement

The studies involving human participants were reviewed and approved by Norwegian Centre for Research Data. Written informed consent to participate in this study was provided by the participants’ legal guardian/next of kin.

## Author Contributions

All authors contributed to the development and design of the study. CF and VR performed testing and data collection. CF, VR, and MT analysed the data and drafted the manuscript.

## Conflict of Interest

The authors declare that the research was conducted in the absence of any commercial or financial relationships that could be construed as a potential conflict of interest.

## Figures and Tables

**FIGURE 1:**
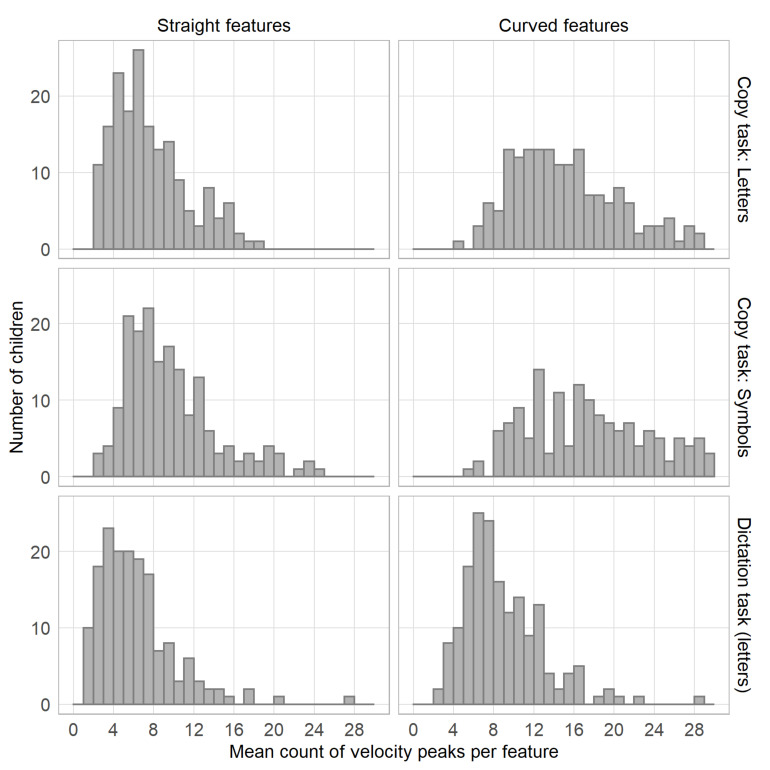
Distribution of velocity peaks.

**FIGURE 2:**
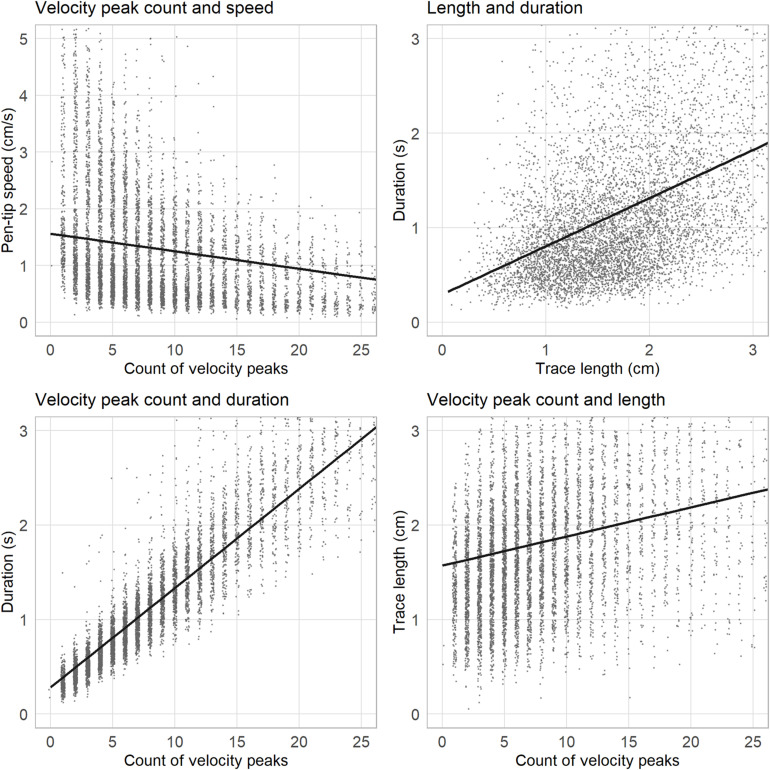
Correlations among fluency, speed, trace length, and duration.

## Footnotes

1 In some cases children struggled to produce garlands. Figure 3 gives an example. To establish that there was no accuracy/fluency trade-off, with children achieving greater fluency by failing to correctly form garlands, we scored each sample for accuracy on two dimensions: the extent to which the pen trace formed loops, and loop direction (up or down). A loop is a segment that goes up, back, down, and forward or down, back, up, and forward. Loops were allowed to overlap, but the next loop had to be displaced to the right. Loops could vary in size. Using these criteria we scored each sample for the extent to which the trace was looped: mainly loops (>75%), some loops (25–75%) and almost no loops (<25%). Disfluency (SNvpd) increased from 63.9 in mainly loops group to 72.5 in the some loops group and to 94.6 for the no loops group. i.e., less accurate forms were associated with less fluent production. There was, therefore, no evidence that students traded accuracy for fluency.

## Appendix: Supplementary Analyses

### Distribution of Velocity Peaks Measure

Figure A1 gives the distribution of our fluency measure, broken down by feature type (straight, curved) and condition (letters and symbols produced in the copy task and letter produced in response to dictation).

### Correlations Among Fluency, Speed, Trace Length, and Duration

The following plot in Figure A2 shows the relationships among these different kinematic measures. Each point on the plot represents that production of one feature by one child, combining data from the copy task (both letters and symbols) and the dictation task. Trace length refers to the total length of the line created as the child produced the feature. Speed is mean speed across production of the feature (trace-length divided by duration). Points are jittered slightly for clarity.

We estimated bivariate correlations among these variables by means of linear mixed effects models with random by-feature and by-child slopes and intercepts. For each pair of measures a model including the predictor variable provided significantly and substantially better fit than model with just random effects [χ^2^(1) > 100, p < 0.001 for all four models]. Correlation (standardised univariate regression) estimates were as follows: Velocity peak count (disfluency) and speed, -0.63, 95% CI [-0.70, -0.55]; length and duration, 0.49 [0.44, 0.54]; velocity peak count and duration, 0.99 [0.96, 1.0]; velocity peak count and length,.37 [0.32, 0.43].
